# Testis-specific ATP synthase peripheral stalk subunits required for tissue-specific mitochondrial morphogenesis in *Drosophila*

**DOI:** 10.1186/s12860-017-0132-1

**Published:** 2017-03-23

**Authors:** Eric M. Sawyer, Elizabeth C. Brunner, Yihharn Hwang, Lauren E. Ivey, Olivia Brown, Megan Bannon, Dennis Akrobetu, Kelsey E. Sheaffer, Oshauna Morgan, Conroy O. Field, Nishita Suresh, M. Grace Gordon, E. Taylor Gunnell, Lindsay A. Regruto, Cricket G. Wood, Margaret T. Fuller, Karen G. Hales

**Affiliations:** 1 Department of Biology, Davidson College, Davidson, NC USA; 2 Departments of Developmental Biology and Genetics, Stanford University School of Medicine, Stanford, CA USA

**Keywords:** *Drosophila melanogaster*, Spermatogenesis, Mitochondria, ATP synthase, Cristae

## Abstract

**Background:**

In *Drosophila* early post-meiotic spermatids, mitochondria undergo dramatic shaping into the Nebenkern, a spherical body with complex internal structure that contains two interwrapped giant mitochondrial derivatives. The purpose of this study was to elucidate genetic and molecular mechanisms underlying the shaping of this structure.

**Results:**

The *knotted onions* (*knon*) gene encodes an unconventionally large testis-specific paralog of ATP synthase subunit d and is required for internal structure of the Nebenkern as well as its subsequent disassembly and elongation. Knon localizes to spermatid mitochondria and, when exogenously expressed in flight muscle, alters the ratio of ATP synthase complex dimers to monomers. By RNAi knockdown we uncovered mitochondrial shaping roles for other testis-expressed ATP synthase subunits.

**Conclusions:**

We demonstrate the first known instance of a tissue-specific ATP synthase subunit affecting tissue-specific mitochondrial morphogenesis. Since ATP synthase dimerization is known to affect the degree of inner mitochondrial membrane curvature in other systems, the effect of Knon and other testis-specific paralogs of ATP synthase subunits may be to mediate differential membrane curvature within the Nebenkern.

## Background

Mitochondrial dynamics, including fusion, division, regulated movement, quality control, and interaction with the endoplasmic reticulum, have been characterized largely in yeast and cultured mammalian cells (reviewed in [1, 2]). Studies in specialized cell types with high energy needs in other organisms, such as neurons and sperm in *Drosophila melanogaster*, also provided early and ongoing insight into molecular mechanisms of mitochondrial shaping [3, 4]. *Drosophila* spermatogenesis involves a concerted developmentally-controlled program of specialized mitochondrial morphogenesis and thus is an ideal system for exploring molecular underpinnings. Mitochondria aggregate during and after male meiosis, subsequently fusing dramatically into two giant mitochondrial derivatives that interdigitate in layers at the early round spermatid stage to generate a large, onion-like, layered spherical structure called the Nebenkern [5–7]. During subsequent spermatid elongation stages, the mitochondrial derivatives within the Nebenkern unfurl and elongate beside the growing flagellar axoneme. Testis-enriched paralogs of more broadly-expressed genes [8] are common and are often identified via genetic analysis as associated with these functions, since null mutations in the testis-enriched paralogs can result in male-sterile but adult-viable mutant phenotypes.

ATP synthase is a large multi-protein complex, found in prokaryotes as well as in mitochondria and chloroplasts of eukaryotes, that catalyzes ATP formation from ADP and inorganic phosphate during cellular respiration and photosynthesis (reviewed in [9]). In mitochondria, the ATP synthase complex typically contains at least 16 subunits divided among the F_1_, F_0_, and peripheral stalk regions. The F_1_ portion extends into the mitochondrial matrix, physically attaching in two ways to the F_0_ portion, which is embedded in the inner membrane: via the F_1_ central stalk that allows rotation, and via the peripheral or stator stalk, which stabilizes the complex. The F_0_ portion rotates in response to proton movement down the gradient established by the electron transport chain, and the resulting torque and conformational changes in the F_1_ portion lead to ATP formation. Defects in the ATP synthase complex in humans can lead to neuromuscular disorders (reviewed in [10]) due to deficiencies in ATP production and overall mitochondrial dysfunction.

As shown mainly in yeast and cultured mammalian cells, the positive membrane curvature at the tips of cristae in the inner mitochondrial membrane is mediated in part by higher-order dimerization and oligomerization of ATP synthase complexes (reviewed in [11–14]). These interactions are separable from the metabolic role of ATP synthase, as some mutants with intact single ATP synthase complexes that cannot dimerize have aberrant cristae structure but can still respire [15, 16]. Cryo-electron microscopy indicates that bovine and yeast ATP synthase complex monomers induce 43° membrane curvature, and that the predicted dimer-induced membrane curvature of 86° matches observed values well [17, 18]. Tomography studies indeed show dimer ribbons corresponding with tight membrane curvature [19].

A logical question is whether regulation of ATP synthase higher-order structures helps to mediate variability in inner mitochondrial membrane conformation across cell types and different conditions. In *Drosophila* spermatogenesis, the large mitochondrial derivatives of the Nebenkern in early round spermatids do not have prominent cristae or sharp positive curvature of the inner mitochondrial membrane; instead the inner and outer mitochondrial membranes appear closely apposed across most of their area and parallel to each other in broad concentric circles of membranes reminiscent of an onion slice (hence early round spermatids are considered to be at the onion stage) [5, 20]. In this way, spermatid wild-type inner mitochondrial membranes resemble yeast inner mitochondrial membranes in cells deficient for ATP synthase dimerization.

Here we report a role for a tissue-specific isoform of ATP synthase subunit d in mediating internal Nebenkern structure within *Drosophila melanogaster* spermatids. We also provide evidence that other tissue-specific ATP synthase subunit paralogs help shape spermatid mitochondria. Subunit d is a major part of the peripheral stalk that stabilizes the F_0_ and F_1_ portions of ATP synthase as they rotate with respect to each other. Subunit d was first described in mammalian mitochondrial ATP synthase [21] and later in yeast [22]. It is not found in bacterial or chloroplast ATP synthase. In *S. cerevisiae*, subunit d is oriented with its amino terminus farthest from the membrane and the carboxy terminus oriented closer to the inner mitochondrial membrane, spanning the distance between the F_0_ and F_1_ portions; while it does not itself span the inner mitochondrial membrane, it interacts via multiple coiled-coil domains with the transmembrane subunit b of the peripheral stalk [23, 24]. We identified an uncharacteristically large ATP synthase subunit d paralog expressed in *Drosophila* testes that is associated with internal Nebenkern structure and that may act via alteration of ATP synthase dimerization. Our work establishes a connection between tissue-specific variants of ATP synthase subunits and tissue-specific internal mitochondrial structure.

## Results

### *knon* mutants show aberrant mitochondrial elongation during spermatogenesis and have faulty Nebenkern internal structure

**Fig. 1:**
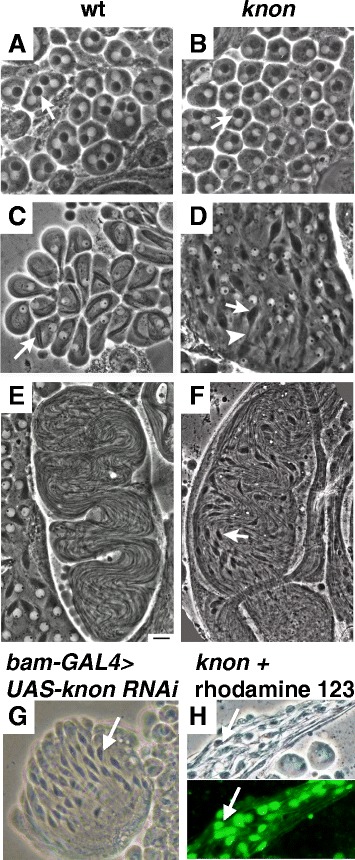
*knon* mutant and knockdown spermatids show aberrant Nebenkern elongation but have an intact membrane potential. Phase-contrast micrographs of live squashed testis preparations showing post-meiotic spermatids from wild-type (**a**, **c**, and **e**) and homozygous *knon* ^*ms(2)1400*^ males (**b**, **d**, **f**, **h**) at the early round (onion) stage (**a**-**b**), the early elongation stage (**c**-**d**), and later in spermatid elongation (**e**-**f**). **g** RNAi knockdown of *knon* in the testis recapitulates the mutant phenotype, with unelongated Nebenkerne (arrow) in nearly mature elongating spermatid cysts. **h** Paired phase-contrast and fluorescence micrographs of an elongating spermatid cyst from a *knon* ^*ms(2)1400*^ */Df(2R)7E* male. Rhodamine 123 uptake by the unelongated mitochondrial derivatives (arrow) in a nearly mature spermatid cyst indicates ongoing respiratory activity. Scale bar 20 μm

**Fig. 2:**
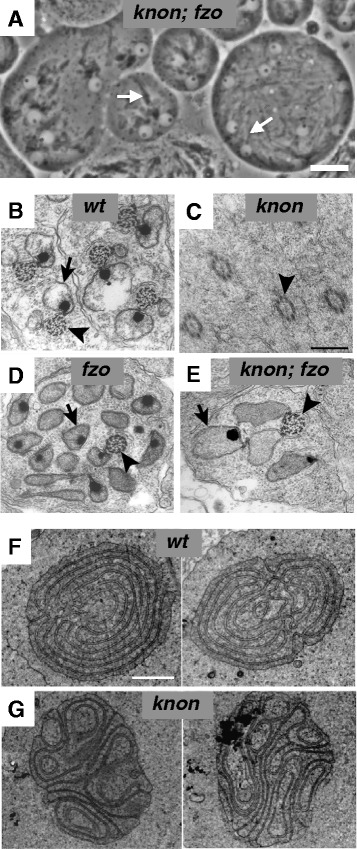
*knon* mutants exhibit aberrant Nebenkern structure and defective mitochondrial elongation that can be suppressed by bypassing mitochondrial fusion. **a** Phase-contrast micrograph of early elongation-stage spermatids from *knon* ^*ms(2)1400*^ *; fzo* ^*1*^ males show some degree of elongation of unfused mitochondria (arrows); compare to Fig. 1c-d. **b**-**e** Cross sections of elongating spermatids viewed by transmission electron microscopy showing axonemes (arrowheads) and elongating mitochondrial derivatives (arrows). Wild-type spermatids (**b**) show two mitochondrial derivatives per axoneme. **c** *knon* ^*ms(2)1400*^ spermatids lack detectable mitochondrial derivatives beside the axoneme. **d** *fzo* ^*1*^ spermatid has multiple mitochondrial derivatives per axoneme resulting from failure of mitochondrial fusion to form the Nebenkern [3]. **e** Spermatid from *knon* ^*ms(2)1400*^ *; fzo* ^*1*^ male shows several mitochondrial derivatives elongating beside the axoneme. Transmission electron micrographs of cross sections of Nebenkerne from wild-type (**f**) and *knon* ^*ms(2)1400*^ (**g**) males. Two examples of each are shown. Internal “onion” structure is disrupted in *knon* ^*ms(2)1400*^ *.* Scale bars 20 μm in **a**, 0.5 μm in **b**-**e**, and 2 μm in **f**-**g**. **b** and **d** adapted from [3]

The *knotted onions* (*knon* ^*ms(2)1400*^) mutant allele of *Drosophila melanogaster* was identified in a screen for recessive male-sterile mutations via mobilization of a marked P element [25]. Subsequent exposure of the mutant chromosome to transposase yielded no revertants of male sterility out of over thirty independent strains that lost *w* ^*+*^-associated eye color, indicating that the spermatogenesis phenotype was likely not associated with an intact P element. Males homozygous for *knon* ^*ms(2)1400*^ were viable but completely sterile and had no individualized, motile sperm. Homozygous *knon* ^*ms(2)1400*^ females were completely viable and fertile. Phase-contrast microscopy of live testis preparations from *knon* ^*ms(2)1400*^ males revealed a failure of the Nebenkern to elongate beside the growing flagellar axoneme (Fig. 1). To distinguish Nebenkern-intrinsic (mitochondrial structure) from Nebenkern-extrinsic (e.g., cytoskeletal or mitochondrial transport defects) sources of elongation failure, we analyzed testes from males double mutant for *knon* and the dynamin-related GTPase *fuzzy onions*, *fzo*, which is required for Nebenkern-associated mitochondrial fusion [3]. We found that *knon* ^*ms(2)1400*^ *fzo* ^*1*^ double mutants exhibit the *fzo* phenotype, wherein many smaller, unfused mitochondria successfully elongate alongside the axoneme, although the elongation appeared to be slightly delayed. This bypass of the elongation defect (Fig. 2, panels a and e) suggests that the *knon* primary defect may be faulty Nebenkern structure at the onion stage that interferes with the ability of mitochondrial derivatives to unfurl for subsequent elongation. Consistent with this idea, transmission electron microscopy (TEM) of *knon* ^*ms(2)1400*^ testes (Fig. 2g) indeed showed aberrant internal Nebenkern structure at the early round spermatid stage (hence the name “knotted onions”). Cross sections of *knon* ^*ms(2)1400*^ elongating spermatid cysts showed axonemes without associated mitochondrial derivatives (Fig. 2c). In contrast, elongating *knon* ^*ms(2)1400*^ *; fzo* ^*1*^ spermatids had multiple (though fewer than in *fzo* alone) mitochondrial derivatives beside elongating axonemes (Fig. 2e). Mitochondria in testes from *knon* ^*ms(2)1400*^ males did have a membrane potential as demonstrated by rhodamine 123 staining (Fig. 1h), suggesting that the phenotype appears to be primarily related to defects in mitochondrial shaping rather than in respiration.

### *knon* is *CG7813*

We originally mapped *knon* by recombination to 2.1 ± 0.7 cM distal of *curved* (polytene region 52D2-9 on chromosome 2R), based on 38 recombinants between *curved* and *welt.* A *w* ^*+*^-marked P element on the *knon* ^*ms(2)1400*^ chromosome mapped near *black*, approximately 30 cM away, confirming its lack of association with the spermatogenesis phenotype. Further recombination mapping placed *knon* 0.67 ± 0.07 cM distal to *Khc* ^*k13219*^ and 0.36 ± 0.07 cM proximal to *Cdk4* ^*k06503*^, suggesting polytene interval 53B-C as the region of interest. *Df(2R)7E* (generated through imprecise excision of *veg* ^*k03402*^), which lacks ~134 kb distal of the *veg* ^*k03402*^ insertion site, failed to complement *knon* ^*ms(2)1400*^, showing a phenotype indistinguishable from *knon* ^*ms(2)1400*^ homozygotes. In contrast, *Df(2R)BSC550* [26] complemented *knon* ^*ms(2)1400*^. Together, the breakpoints of *Df(2R)7E* and *Df(2R)BSC550* defined the *knon* region as including two genes, *Sema2a* and *CG7813* [27]. *Sema2a* is expressed in many tissues [28] and known to function in the nervous system [29]. FlyAtlas data indicate that *CG7813* is expressed highly in the testis and at near-zero levels in adult carcass (minus the germline) and all other tissues tested except larval fat body, in which the larval gonad is embedded [28]. modENCODE RNAseq data indicate that *CG7813* is expressed highly in wandering third instar larvae and adult males but not at all in adult females [30], consistent with expression only in the male reproductive tract. Together these data suggested *CG7813* as the best candidate for *knon*.

**Fig. 3:**
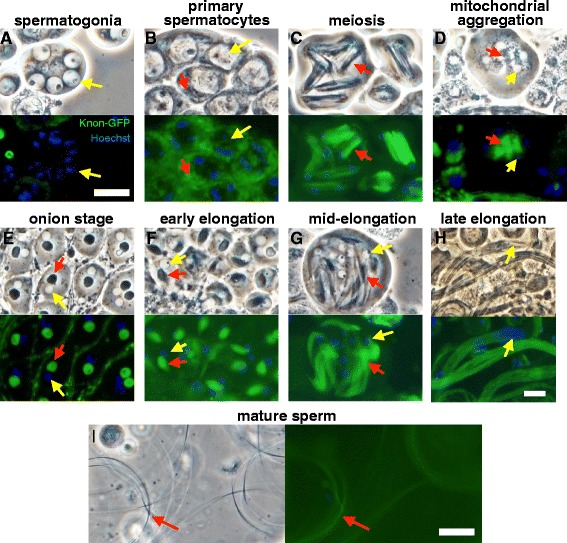
Knon-GFP rescues the mutant phenotype and localizes to mitochondria in primary spermatocytes and post-meiotic spermatids. Phase-contrast and paired fluorescence images of cells from testes of *knon* ^*ms(2)1400*^ */Df(2R)7E*; *knon-GFP*/+ males, stained with Hoechst. Knon-GFP (green) was not detectable in spermatogonia (**a**) but was associated with phase-dark mitochondria (red arrows) in all later stages (**b**-**i**). Yellow arrows indicate nuclei; red arrows indicate mitochondria-associated Knon-GFP. In primary spermatocytes (**b**), Knon-GFP-labeled mitochondria were small and diffuse in the cytoplasm. In meiotic cells (**c**), Knon-GFP-marked mitochondria associated with the spindle; immediately after meiosis (**d**) mitochondria aggregated beside each nucleus and were associated with a strong Knon-GFP signal. The mitochondrial derivatives within the Nebenkern at the onion stage (**e**, red arrow) and during early- (**f**) and mid-elongation (**g**) showed unambiguous Knon-GFP localization. In nearly mature elongating spermatid cysts (**h**), elongated flagella appear wild type with no clumped mitochondrial derivatives (compare to Fig. 1). Knon-GFP is evenly distributed throughout the elongated spermatids. After sperm individualization, motile sperm (**i**) retain detectable Knon-GFP (red arrow). The apparent syncytial nature of some cells at earlier stages is an artefact of the preparation—ring canals connecting cells in a cyst are commonly burst open by the pressure from a cover slip. These localization results are identical to those seen when Knon-GFP is expressed in a wild-type background (not shown). Scale bars in **a**-**g**, **h**, and **i** are 20 μm

Rescue experiments and phenocopy by RNAi knockdown confirmed *CG7813* as the gene mutated in *knon* ^*ms(2)1400*^. Transgenic male flies homozygous for *knon* ^*ms(2)1400*^ and carrying an exogenous copy of *CG7813* under its own regulatory regions were fertile and exhibited wild-type mitochondrial shaping and elongation during spermatogenesis (Fig. 3). Knocking down *CG7813* in the germline using the *bam-GAL4* driver [31] and the *P[GD12291]v22566 CG7813* RNAi insertion from the Vienna Drosophila Resource Center [32] resulted in sterile males with large unelongated masses of mitochondria at the base of each elongating post-meiotic spermatid, similar to the *knon* ^*ms(2)1400*^ phenotype (Fig. 1). Neither the RNAi transgene alone nor the *bam-GAL4* transgene alone had any detectable effect on spermatogenesis or male fertility (not shown). We amplified and sequenced the coding region of *CG7813* from *knon* ^*ms(2)1400*^ homozygotes and found no difference compared to the reference sequence [27], while an upstream region was not amplifiable in *knon* ^*ms(2)1400*^. It is therefore likely that the *knon* ^*ms(2)1400*^ lesion is in an upstream regulatory region that was disrupted, possibly by P-element insertion and subsequent excision during the original mutant screen [25].

The MiMIC (Minos Mediated Integration Cassette) insertion line *Mi[MIC]CG7813* ^*Mi10492*^ [33, 34] is a hypomorphic allele of *knon (CG7813)* and is henceforth referred to as *knon* ^*Mi10492*^. The *knon* ^*Mi10492*^ insertion is in the coding region of *CG7813* between codons 554 and 555 [27] and is therefore predicted to truncate the last 180 amino acids of Knon. Since the element is in reverse orientation with respect to the *knon* coding sequence, it does not provide a GFP tag to the truncated protein. Homozygous *knon* ^*Mi10492*^ males were fertile but at slightly reduced levels, giving rise to 85% as many progeny as wild-type males (*n* = 56; *P* = 1.29x10^-6^), and show incompletely penetrant mild mitochondrial elongation defects (not shown). Males with *knon* ^*Mi10492*^ in *trans* to either *Df(2R)7E* or *knon* ^*ms(2)1400*^ were completely sterile with a mitochondrial elongation defect in spermatids similar to that in *knon* ^*ms(2)1400*^ homozygotes, further confirming that the original *knon* ^*ms(2)1400*^ phenotype is due to a lesion in *CG7813.*

### Knon is a testis-enriched ATPsynD paralog with large C-terminal extension and associates with spermatid mitochondria

The *knon* gene encodes a 734 amino acid protein with an amino terminal region 32% identical and 50% similar to *Drosophila* ATPsynD (178 amino acids), the broadly-expressed canonical d subunit of the ATP synthase complex. Subunit d is part of the peripheral (or stator) stalk connecting the F_0_ and F_1_ portions of the complex. Knon, a testis-enriched paralog of subunit d, has 532 additional amino acids at its carboxy terminus beyond the region of homology. BLASTp and tBLASTn searches revealed that this C-terminal region was not homologous to other characterized gene products [35]. Curiously, in this C-terminal region 28% of the residues are either glutamic acid or lysine, making it highly charged, and there are no predicted transmembrane domains or other motifs. The IUPred tool [36] predicts that most of this region is intrinsically unstructured.

Knon-GFP expressed under the control of endogenous *knon* regulatory sequences rescued the mutant phenotype (confirming functionality and relevance of localization) and was associated with mitochondria during later stages of spermatogenesis (Fig. 3). Knon-GFP was not detectable in the apical testis, which contains the stem cell populations and mitotically proliferating spermatogonia. The protein was detectable starting in spermatocytes during chromosome condensation. The timing of transition from absence of Knon-GFP to abundance was consistent with established patterns of transcription in primary spermatocytes [37]. Knon-GFP co-localized with phase-dark mitochondria throughout spermatogenesis, associating with meiotic spindles, post-meiotic aggregating mitochondria, and Nebenkerne in the onion stage. Knon-GFP persisted on mitochondrial derivatives during and after mitochondrial elongation in elongated spermatid cysts. Mature, individualized wild-type sperm also contained the protein.

### Exogenous expression of Knon in flight muscle alters the ratio of ATP synthase complex dimers to monomers but does not affect gross mitochondrial shaping or function

**Fig. 4:**
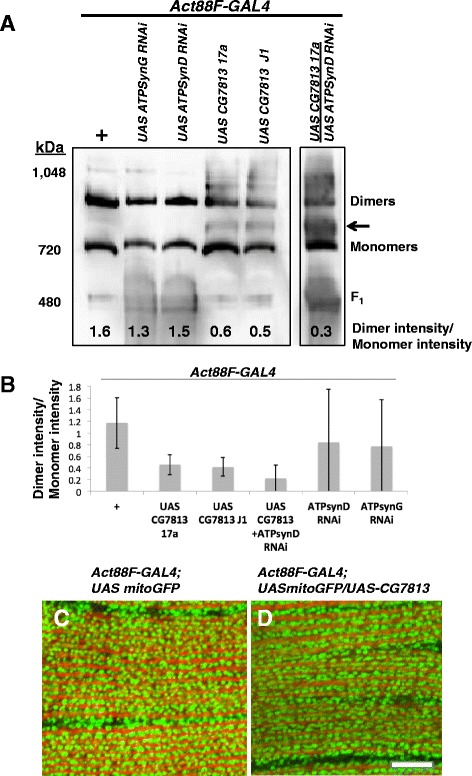
Knon expression in flight muscle alters ATP synthase dimer:monomer ratio but not gross mitochondrial morphology. **a** Immunoblot using anti-ATP synthase α subunit antibodies on a BN-PAGE gel of mitochondrial isolates from flight muscle of the indicated genotypes. The *Act88F-GAL4* flight muscle driver was used as a control (first lane), as well as to drive exogenous Knon (CG7813) expression from two independent transgene insertions (fourth and fifth lanes), RNAi knockdown constructs of ATP synthase subunits g (second lane) and d (third lane), or simultaneous exogenous CG7813 expression and knockdown of ATP synthase subunit d (sixth lane). Dimer/monomer intensity ratios are indicated at bottom. Expressing Knon (CG7813), either with or without concurrent knockdown of the broadly-expressed ATPsynD, reduced the amount of ATP synthase dimers relative to monomers and gave rise to detectable bands of slightly larger size (arrow) than the typical monomer bands. **b** Quantification of dimer/monomer band intensity ratios from multiple biological replicates (n = 3 for *+*, *UAS CG7813 17a*, and *UAS CG7813 J1*; *n* = 4 for *UAS CG7813* + ATPsynD RNAi; *n* = 2 for ATPsynD RNAi and ATPsynG RNAi). Error bars represent 95% confidence intervals. **c**-**d** Confocal images of *Drosophila* flight muscle from *Act88F-GAL4; UAS-mitoGFP* (**c**) flies and *Act88F-GAL4; UAS-mitoGFP/UAS-CG7813* flies (**d**). Scale bar 10 μm

Because the ATP synthase d subunit is located at the interface of bound ATP synthase complexes in a dimer, we hypothesized that the extended C-terminal domain of Knon might favor the monomeric ATP synthase configuration by a steric mechanism. To test whether incorporation of Knon into ATP synthase inhibits dimerization of the complex, we exogenously expressed full-length Knon in flight muscle with the *GAL4-UAS* system [38] and analyzed ATP synthase complexes by blue native polyacrylamide gel electrophoresis (BN-PAGE) and immunoblotting. (Testis tissue is not amenable to this approach since each dissected testis includes cells at multiple stages, and since the dramatically varying mitochondrial conformations in spermatids make isolation of the organelle impractical.) We generated flies carrying the wild-type *knon* coding region, with or without a C-terminal GFP tag, downstream of the *UAS* regulatory region, and crossed these to flies with the flight muscle *Act88F-GAL4* driver. We confirmed by fluorescence microscopy robust *Act88F-GAL4*-induced Knon-GFP expression in flight muscle (not shown). By BN-PAGE and immunoblotting using an antibody specific for ATP synthase subunit α, we examined the internal ratio of ATP synthase dimers to monomers in flies with *Act88F-GAL4* alone compared to *Act88F-GAL4* driving Knon expression from each of two independently-inserted *UAS-knon* transgenes (Fig. 4). Quantification of band intensity indicated a statistically significant (*P* < 0.05) shift toward monomers when Knon is expressed, compared to a control. Such a shift was not seen when we used the *Act88F-GAL4* driver to drive RNAi constructs knocking down the broadly-expressed ATPsynD or ATPsynG, though these conditions led to a more pronounced F_1_ band and a smear at lower molecular weights suggesting increased dissociation of the complex into F_0_ and F_1_ portions and subsequent degradation. To test whether exogenous Knon would exert a stronger effect if less of the paralogous ATPsynD was present, we simultaneously expressed Knon and knocked down ATPsynD; the shift toward ATP synthase monomers was similar to expressing Knon alone (Fig. 4a), although with the more pronounced lower molecular weight smear as seen in the knockdown alone. In each case, when Knon was expressed in flight muscle, the BN-PAGE immunoblot also showed bands slightly heavier than the typical monomer bands (Fig. 4a, arrow), perhaps indicating monomers of the ATP synthase complex that incorporated Knon (with its extra 532 amino acids) instead of ATPsynD. While native gels do not allow for precise calculation of molecular mass from distance traveled, the position of the extra band could plausibly represent monomers with an extra 62 kD.

To ask whether the Knon-mediated shift toward more ATP synthase monomers was associated with altered flight muscle mitochondrial morphology, we examined by confocal microscopy tissue from flies expressing Knon in flight muscle along with a separate mitochondrially-targed GFP (mitoGFP) [39] (Fig. 4c-d). The mitoGFP localization was roughly similar between flies with and without exogenously expressed Knon, suggesting no gross alteration in mitochondrial number or size. Consistent with the lack of obvious effect on mitochondrial structure, flies expressing Knon in flight muscle were viable and able to fly, though there may be subtle effects at the levels of tissue architecture and flight ability that our experiments did not detect.

### Other testis-enriched paralogs of ATP synthase subunits diverged at different times within insect lineages and have roles in spermatid mitochondrial dynamics

In addition to subunit d, the *D. melanogaster* genome includes pairs of paralogs encoding ATP synthase subunits F6, b, and g (which are in the peripheral stalk), as well as ε and β (in the F_1_ portion of the complex) [27]. Expression data from modENCODE [30] and FlyAtlas [28] consistently indicate strong testis enrichment for one gene within each paralogous pair. Similar to subunit d, for subunit F6 the testis-enriched predicted gene product (147 amino acids) is significantly larger than that of the broadly-expressed paralog (106 amino acids). Conversely, for subunit b the testis-enriched predicted gene product (152 amino acids) is significantly smaller than that of the broadly expressed paralog (243 amino acids).

**Fig. 5:**
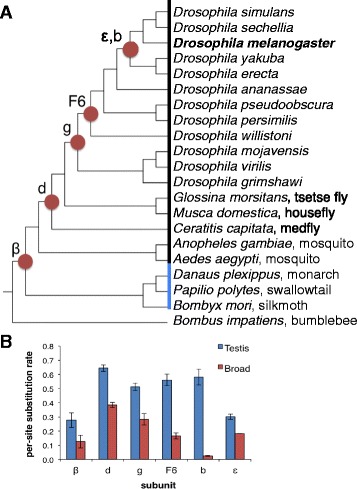
Phylogeny and evolution of Knon and other ATP synthase subunit paralogs in insects. **a** The six ATP synthase subunits encoded by two paralogs in *D. melanogaster* have widely varying phylogenetic origins, determined by comparisons among insect genome sequences (black, Diptera; blue, Lepidoptera) deposited in NCBI. Circles indicate the most recent common ancestor that contained both paralogs. **b** Testis-enriched subunits have acquired more amino acid substitutions relative to a recent outgroup than broadly expressed subunits. The testis-specific ortholog of each subunit was inferred based on sequence similarity to *D. melanogaster* orthologs, which were in all cases unambiguous. The average per-site amino acid substitution rate for each subunit type relative to the most recent outgroup of the gene duplication was calculated from a Clustal Omega alignment [76]. Error bars indicate standard deviation among the species analyzed

We searched other sequenced genomes and found that the genes encoding ATP synthase subunits diverged into paralogous pairs at different times in insect lineages (Fig. 5). Maximum likelihood phylogeny analysis (not shown) confirmed that the proteins encoded by a given pair of paralogs across species clustered in two groups around each of the differentially expressed *D. melanogaster* paralogs. In each case, the grouping that included the *D. melanogaster* testis-enriched paralog showed a much higher per-site amino acid substitution rate (Fig. 5b), suggesting that they are evolving faster, consistent with previous observations [8]. For each subunit, the two paralogs were not in close proximity in the genome, and in no case was there synteny in their respective regions. For the paralogs encoding the ATP synthase F6 subunit, the testis-enriched version lacks introns, consistent with retroduplication as a possible duplication mechanism [40]. For subunits b and g, the testis-enriched paralog contains introns but fewer than in the broadly-expressed version, consistent with a possible retroduplication mechanism involving alternatively spliced transcripts as the gene duplication source [41].

**Fig. 6:**
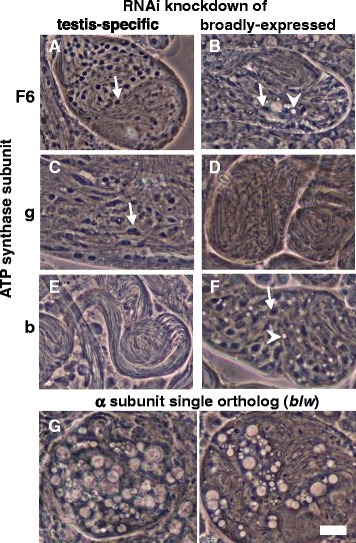
RNAi knockdown of other ATP synthase subunits in the testis. **a**-**f** *bam-GAL4*-driven UAS-RNAi testis knockdown of ATP synthase subunit paralogs that show either testis-enriched expression (left column) or broad expression across multiple tissues (right column). Two of the three testis-enriched paralogs of peripheral stalk subunits—F6 (**a**) and g (**c**), but not b (**e**)—show a similar knockdown phenotype to *knon*, with failure of mitochondrial elongation (arrows) in spermatid cysts that have nearly fully elongated, along with individualization failure, a common secondary effect of spermatid morphology defects [6]. Knockdown of broadly-expressed paralogs of subunits F6 (**b**) and **b** (**f**) results in aberrant mitochondrial elongation along with vacuolar inclusions (arrowheads), whereas knockdown of broadly-expressed subunit g (**d**) results in elongating spermatid cysts of wild-type appearance. **g** Testis knockdown of the sole ATP synthase α subunit ortholog (*bellwether*), part of the F_1_ portion of ATP synthase, resulted in large vacuolar inclusions and degradation of elongating spermatid cysts. Two representative cysts are shown. Scale bar 20 μm for **a**-**g**. For each genotype, >80 spermatid cysts were visualized from >6 dissected males

Inducing RNAi knockdown of testis-specific ATP synthase subunits F6 and g in the *Drosophila melanogaster* testis using the *bam-GAL4* driver led to mitochondrial elongation defects similar to those seen in homozygous *knon* males (Fig. 6), while knockdown of the broadly-expressed paralogs showed more subtle effects. Conversely, knockdown of the more recently diverged testis-specific b subunit did not affect spermatogenesis, while the broadly-expressed subunit b appears to retain an essential role in mitochondrial shaping during spermatogenesis. In contrast to these results, testis knockdown of the sole ortholog of the ATP synthase α subunit (*bellwether*) [42], which is in the F_1_ portion and is crucial for ATP synthesis, resulted in degenerating spermatid cysts filled with large, prominent vacuolar inclusions. In general, these results are consistent with the premise that the ATP synthase complex controls high-level mitochondrial architecture in developing spermatids through a mechanism separable from ATP synthesis.

## Discussion

### The Knon ATP synthase subunit d paralog is required for normal Nebenkern morphology

We found a role for Knon, an uncharacteristically large paralog of ATP synthase subunit d, in internal shaping of mitochondrial membranes in the Nebenkern during *Drosophila* spermatogenesis. Male flies lacking functional Knon were sterile and showed unusual membrane curvature and faulty internal structure within the Nebenkern (Fig. 1), with subsequent entanglement of the two unfurling mitochondrial derivatives and aberrant mitochondrial elongation as the flagellar axoneme grows. Simultaneously preventing mitochondrial fusion in a *knon*; *fzo* double mutant suppresses the *knon* single mutant elongation defect. This result indicates that the *knon* phenotype is due to the formation of an aberrant Nebernkern structure that cannot elongate, rather than defects at later stages. Knon is expressed only in the testis [28], and the protein is detected on mitochondria from meiosis through spermatid elongation (Fig. 3), consistent with the timing of dramatic mitochondrial membrane reorganization.

The effect of *knon* on mitochondrial shaping is likely at the level of mitochondrial dynamics and not resulting from respiratory deficiency, since 1) the mitochondrial membrane potential remains high in *knon* mutant spermatids; 2) other energy-requiring events of spermatid development, such as axoneme elongation and nuclear shaping, proceed; 3) disrupting ATP synthesis by knocking down the sole ATP synthase α subunit ortholog in the testis leads to tissue degeneration; and 4) in other systems, the effects of ATP synthase on organelle morphology and respiration are separable [15, 16].

**Fig. 7:**
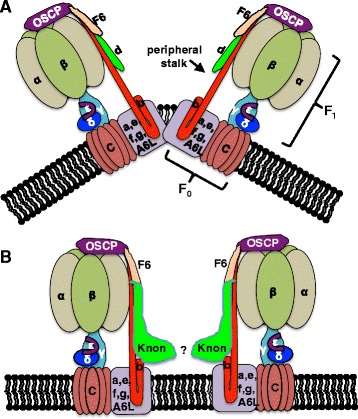
Model for alteration of ATP synthase complex dimerization by Knon. **a** Schematic representation of ATP synthase complex dimerization as mediated by various subunits in the peripheral stalk and F_0_ portion. **b** Steric interactions of the large Knon C termini might inhibit dimerization or enable dimerization at a shallower angle

In many cell types, dimerization of ATP synthase complexes with their axes at an angle of 86° is important for determining sharp positive curvature of the inner mitochondrial membrane at cristae tips. In the Nebenkern, however, the majority of the inner mitochondrial membrane is closely apposed to the outer mitochondrial membrane and shows very shallow curvature. The unusual membrane configuration in the Nebenkern represents accumulation of inner and outer membrane material that is subsequently needed during mitochondrial elongation [20]. We hypothesized that, normally, Knon expression during spermiogenesis may inhibit or alter ATP synthase dimerization to facilitate the more shallow curvature of the bulk of the inner mitochondrial membrane as the Nebenkern forms. In *knon* mutant spermatids, because the broadly-expressed paralog of subunit d (CG6030) is also expressed in the testis [28, 30], Nebenkern membranes may show aberrant sharp curvature from ATP synthase dimerization (Fig. 7).

### Effects of Knon on ATP synthase dimerization

In yeast and mammalian cells, ATP synthase subunits e, g, and b are known to mediate dimerization of ATP synthase complexes and contribute to the sharp curvature of inner mitochondrial membranes at cristae tips [43, 44]. *S. cerevisiae* cells mutant for subunit e or subunit g, normally found in the membrane-embedded F_0_ portion of the complex, lack higher order ATP synthase dimers and have onion-like mitochondrial structures, reminiscent of the fly Nebenkern, that lack the tight inner membrane curvature normally found in cristae [45–47]. Subunit b in the peripheral stalk is also important for dimerization of the ATP synthase complex, and it can homodimerize on its own [48, 49]. In yeast with a truncated subunit b missing the transmembrane portion, no ATP synthase dimers form, and inner mitochondrial membranes lack cristae [44].

Peripheral stalk components farther away from the membrane also contribute to ATP synthase dimerization. Subunit h (F6 in metazoans), in the peripheral stalk, can homodimerize and stabilize dimers in yeast even if subunits e and g are missing [50, 51]. Cryo-electron microscopy of ATP synthase dimers from the alga *Polytomella* indicates that peripheral stalk components from adjacent ATP synthase monomers intertwine at a position 80 angstroms away from the inner mitochondrial membrane [52], consistent with the positions of subunits d and h/F6. The broadly-expressed *D. melanogaster* subunit d (CG6030) has 178 amino acids and is expressed in all tissues, although at lower levels in testes [28]. Orthologs in humans, mice, *C. elegans*, and budding yeast are similar in size, with 161, 161, 191, and 174 amino acids, respectively. In contrast, Knon has 734 amino acids as a result of a large C-terminal extension. The crystal structure of bovine subunit d suggests that its carboxy terminus extends from the middle of the peripheral stalk toward the membrane and embedded F_0_ portion [23], consistent with the idea that Knon’s extra large domain in that position may alter the interactions between ATP synthase complex monomers.

Exogenous expression of Knon in flight muscle indeed reduces dimerization of ATP synthase (Fig. 4). Expression of Knon in the absence of testis-specific versions of subunits F6, g, and b may not, however, allow for assembly dynamics of a full testis-specific ATP synthase complex. We therefore cannot rule out the possibility that in wild-type spermatids, Knon along with the other testis-specific paralogs may help mediate ATP synthase dimerization at a shallower angle. Dimerization of ATP synthase complexes at a shallower angle has been seen in *Tetrahymena* [53]. ATP synthase dimerization in the testis is difficult to assess directly because of tissue heterogeneity and technical constraints.

Truncated Knon^Mi10492^ lacking the carboxy-terminal 180 amino acids is partially functional, suggesting that 554 amino acids (plus any encoded on the inserted element before the first stop codon is encountered), are sufficient for Knon to perform its function when threshold levels of protein are present. These data are consistent with the carboxy-terminal extension either providing steric hindrance that blocks dimerization or promoting unique interactions that underlie dimerization at a different angle.

### Roles for other testis-enriched ATP synthase paralogs in spermatid mitochondrial shaping, and implications of different divergence times

Three other subunits of ATP synthase in the peripheral stalk and/or at the putative dimerization interface (F6, g, and b), and two subunits in the F_1_ portion (β and ε), appear to be encoded by broadly-expressed and testis-specific paralogs in the *Drosophila melanogaster* genome. We showed via RNAi knockdown experiments that the testis-specific paralogs of subunits F6 and g are required for proper Nebenkern dynamics during spermatogenesis, and that these phenotypes differed from the outcome of disrupting ATP synthesis via knockdown of the sole α subunit ortholog. The testis-enriched F6 subunit, like the Knon subunit d, is larger than the broadly-expressed version, while the two g subunit paralogs are similar in size. The testis-enriched g subunit therefore might not provide a steric hindrance to dimerization of the complex but might instead promote assembly of the testis-enriched large d and F6 subunits into the complex, or promote interactions between adjacent ATP synthase complexes at a shallower angle at the base of the peripheral stalk.

For subunit b, the RNAi transgenic line targeting the testis-enriched paralog may not be sufficiently inducible to show an effect, or alternatively the testis-enriched subunit b might truly be not required for spermatid morphogenesis. Consistent with our data, another group recently showed that knockdown of the broadly-expressed ATPsynB results in male infertility and a lack of mature sperm, though they did not examine the mitochondrial phenotype [54]. Dispensability of the testis-specific subunit b would be consistent with its relatively recent gene duplication (Fig. 5), such that there has been less time for specialization of the two paralogs compared to the other paralogous pairs.

The mechanism of differential gene regulation between the paralogs of peripheral stalk ATP synthase subunits is unknown. Notably, the paralogous ATP synthase β subunits were duplicated earlier than the others, and a recent study showed that the broadly-expressed Drosophila ATPsynβ is normally repressed in germ cells by the hairpin RNA pathway [55]. Derepression of ATPsynβ via knockout of the appropriate hpRNA causes defects in germ cell differentiation and male sterility [55]. These findings suggest that the ATPsynβ paralogs have functionally diverged and have evolved RNA-based regulatory mechanisms to favor the expression of one paralog over the other in the testis. The mechanisms of regulation of the other paralogous pairs are yet to be determined.

### ATP synthase, mitochondrial shaping, and tissue differentiation

In mammalian systems the ultrastructural organization of the inner mitochondrial membrane can vary among tissue types and in response to metabolic state or developmental cues (reviewed in [14]). Expression of subunit e, important for dimerization, can be triggered by altered nutrient and oxygen levels [56, 57]. In differentiating cardiomyocytes, mitochondria undergo structural changes concomitant with ATP synthase dimer stabilization by an accessory factor [58, 59]. In the syncytiotrophoblast outer layer of the placenta, reduced ATP synthase dimerization accompanies a lack of cristae, despite normal ATP production [60]. Specific mutations in the mitochondrially-encoded ATP6 subunit found in Leigh syndrome patients are associated with higher levels of ATP synthase dimerization and decreased turnover of the complex [61].

The *Drosophila* ovary requires broadly-expressed ATP synthase components for germ cell differentiation [62], possibly associated with mitochondrial morphology and not respiration, because knockdowns of other respiratory complexes did not show the same effect. Germline-specific ATP synthase subunits in *C. elegans* appear required for tissue differentiation [63], though no mitochondrial structural aspects were examined. In trypanosomes, different ATP synthase subunit c paralogs are encoded in the genome; it is not yet known whether these gene products influence mitochondrial function and morphology in different life cycle stages [64], though subunit c is not positioned to influence dimerization of the complex directly. Our findings in the *Drosophila* testis therefore elucidate the first known instance in which tissue-specific ATP synthase subunits underlie tissue-specific regulation of mitochondrial shaping.

## Conclusions

We describe the first evidence connecting tissue-specific mitochondrial shaping with the expression and function of tissue-specific ATP synthase subunits. A testis-specific variant of ATP synthase subunit d in *Drosophila* is required for Nebenkern morphology and appears to alter dimerization of ATP synthase complexes. Testis-specific paralogs of other ATP synthase subunits also appear to have a role. Our data, showing the role of a cell biological process in the context of tissue morphogenesis, contribute to the growing knowledge of ATP synthase’s role in mediating membrane conformation, beyond its canonical role in ATP production. Future work with tissue-specific ATP synthase subunit paralogs in *Drosophila* may explore their sub-mitochondrial localization to test localization in areas of shallower inner mitochondrial membrane curvature; further work should also explore whether the testis-specific paralogs together form a unique complex, and whether individual subunits can be substituted between the different forms of the complex.

## Methods

### Fly stocks, husbandry, and fertility tests

Flies were raised on standard cornmeal molasses medium or instant potato flake medium (Ward’s Scientific) at 25 °C for all crosses except for those combining *GAL4* and *UAS* transgenes, which were maintained at 29 °C. Multiply marked stocks, transposable element insertions, the Δ2-3 transposase-encoding stock, the *Act88F-GAL4* driver stock, and all deficiencies except *Df(2R)7E* were obtained from the Bloomington *Drosophila* Stock Center. *Oregon R* was used as the wild-type strain. Flies with transgenic UAS-RNAi constructs were obtained from the Vienna Drosophila Resource Center. The *bam-GAL4* driver stock was a gift from Yukiko Yamashita. Most fertility tests were performed by mating individual males with three *Oregon R* virgin females and assessing the presence of larvae after five days. For quantifying fertility of *knon* ^*Mi10492*^ allelic combinations, 56 age-matched males of each genotype were placed individually in vials with three age-matched *Oregon R* virgin females; pupae and eclosed offspring from vials in which the males survived were counted after fifteen days. Data were analyzed by *T* test.

### RNAi knockdown

We obtained all RNAi transgenic lines from the Vienna Drosophila Resource Center [32], listed here according to their targets:

- *P[GD12291]v22566* (*knon;* CG7813; testis-specific subunit d)
- *P[KK1096241]v104818* (CG12027; testis-enriched subunit F6)
- *P[KK108581]v107826* (CG4412; broadly-expressed subunit F6)
- *P[GD12493]v35387* (CG7211; testis-enriched subunit g)
- *P[KK108323]v107311* (CG6105; broadly-expressed subunit g)
- *P[GD1688]v6521* (CG17300; testis-enriched subunit b)
- *P[GD6129]v14211* (CG8189; broadly-expressed subunit b)
- *P[GD11030]v34663* (CG3612 (*bellwether*); sole ortholog of subunit α)

For knockdown in the testis, each RNAi line was crossed to a *bam-GAL4* driver line [31]. Testes of F1 males were dissected and imaged as described below. For RNAi knockdown in flight muscle, each relevant UAS-RNAi line was crossed to the *Act88F-GAL4* third chromosome driver line (Bloomington Drosophila Stock Center).

### Microscopy

For light microscopy, testes were dissected in TB1 buffer (7 mM K_2_HPO_4_, 7 mM KH_2_PO_4_ (pH 6.7), 80 mM KCl, 16 mM NaCl, 5 mM MgCl_2_, 1% PEG-6000) and opened with forceps to allow cells to form a monolayer under pressure of a cover slip. Co-labeling of DNA and polarized mitochondria was performed by incubating intact testes in TB1 supplemented with 5% DMSO, 2.5 μg/mL Hoechst, and 10 μg/mL rhodamine 123 [65] for ~10-15 min in the dark. The testes were washed three times with TB1 to remove residual stain and then torn open on a polylysine-coated slide prior to imaging. Phase-contrast and epifluorescence microscopy were performed on a Nikon Eclipse E600W, Olympus B201, or Olympus BX60 microscope, and images were captured with a Nikon Coolpix 4500 or Olympus DP80 camera.

For confocal microscopy, thoraces of adult males and females were dissected to obtain flight muscle tissue. Thoraces were fixed in 4% paraformaldehyde with PBS, washed 3x with PBT (0.2% TritonX), and stained overnight with tetramethylrhodamine-conjugated phalloidin in PBT at a concentration of 8 μM. Whole thoraces were mounted on a slide with mounting media and sealed with a coverslip and clear nail polish. The flight muscle extruded from the exoskeleton after pressing and sealing the coverslip. Samples were observed with a Nikon 90i confocal microscope and a Plan Apo TIRF 100X/1.45 oil objective. Images were captured and overlaid using the confocal software EZ-C1.

For transmission electron microscopy, testes were dissected in TB1 and immediately placed in fixative (2% glutaraldehyde, 1% paraformaldehyde, 0.1 M sodium phosphate or sodium cacodylate buffer (pH 7)). After overnight fixation, samples were washed in 0.1% phosphate or cacodylate buffer for 15 min and stained with 1% osmium tetroxide in the same buffer for two hours. Testes were then washed three times in water, stained for 1 h in 1% uranyl acetate, washed three times in water and dehydrated through an ethanol series (30%, 50%, 70%, 95%, 100%). After five minutes in 1:1 ethanol:propylene oxide and five minutes in propylene oxide, samples were embedded in Spurr’s resin and polymerized overnight at 60 °C. Thin sections (80-90 nm) were cut with a Reichert-Jung microtome, placed on Formvar-coated slot grids and examined on a Phillips 410 transmission electron microscope.

### Recombination and deficiency mapping

Virgin females of genotype *knon/ al dp b r cn vg c a px bw mi sp* were crossed to *al dp b pr Bl c px sp/CyO* males. F1 males that carried *Bl* along with other markers indicating single recombination events on the second chromosome were selected for each interval and crossed individually to *w; knon/CyO* females to score both *knon* and the *w* ^+^-marked P element, and to make stocks of the recombinant chromosome with *CyO*. For finer mapping, *knon/c wt px* females were crossed to *al dp b pr Bl c px sp/CyO* males, and F1 male recombinant progeny in the *c-px* interval were individually crossed to *knon/CyO* to score *knon* and make stocks of recombinant chromosomes. The *welt* (*wt*) marker was scored in homozygotes in each of the recombinant stocks. For deficiency mapping, *knon/CyO* flies were crossed to selected 2nd chromosome deficiency lines balanced over *CyO*, and the Cy^+^ male offspring were tested for fertility and dissected as described above to assess testis subcellular phenotype.

### Generation of transgenic lines

A *knon* transgene with endogenous regulatory sequences was generated by amplifying a ~5 kb genomic region containing *CG7813* along with ~1.8 kb upstream and ~0.5 kb downstream, with primers 5’ ATATATGGTACCGCCATCAGCCAGCGAGCTT 3’ (forward) and 5’ GCATACCGCGGGGCAACACTTTTCGAACGG 3’ (reverse), and cloning the fragment into the pCaSpeR4 vector [66] using restriction sites KpnI and SacII. Primers were purchased from Integrated DNA Technologies (Coralville, IA). A separate *knon-GFP* transgene was created to include the ~1.8 kb upstream region and *knon* coding region through the penultimate codon*,* with *GFP* (GFPmut3* variant with S2R, S65G, S72A [67]) ligated in-frame 3’ to the coding region at the SacII restriction site. To create *UAS-knon* and *UAS-knon-GFP* constructs, the *knon* and *knon-GFP* constructs described above were used as PCR template in two-step splicing by overlap extension. The single *knon* intron was removed by amplifying the two exons of the gene in separate PCR reactions, using Phusion High-Fidelity PCR Master Mix with HF Buffer (New England Biolabs Mo531S). The two products were then pooled in a third reaction, where they annealed and amplified to form a product with sequence identical to the cDNA. The inserts were cloned into pUAST ([38]; obtained from the *Drosophila* Genomics Resource Center, Bloomington, IN) using KpnI and XbaI (New England Biolabs). DNA sequencing to check constructs was performed by Retrogen (San Diego, CA), and DNA sequence analysis was performed in ApE [68]. Constructs were injected into *w* ^*1118*^ embryos by Rainbow Transgenics (Camarillo, CA). *w* ^*+*^ F1 offspring were outcrossed to *Kr/CyO; D/TM6C* to make stocks and map insertions. *knon* and *knon-GFP* transgenes inserted on the third chromosome were crossed into the *knon* mutant background.

### BN-PAGE and immunoblotting of flight muscle mitochondria

Mitochondria were extracted from fly thoraces using a protocol modified from [69]. Fifteen thoraces from flies of each genotype were crushed in 50 μL mitochondrial isolation medium (MIM: 250 mM sucrose, 10 mM Tris-HCl, pH 7.4, 0.15 mM MgCl_2_) using a plastic pestle, then spun twice at 500 x g for 5 min at 4 °C to remove debris. The supernatant was spun at 5,000 x g for 5 min at 4 °C to pellet mitochondria. The pellet was subjected to the Life Technologies NativePAGE Novex® Bis-Tris Gel System protein isolation protocol for BN-PAGE. Protein pellets were resuspended in 25 μL of 1x Native PAGE sample buffer (Invitrogen) containing 1% digitonin. Samples were incubated on ice for 15 min and centrifuged at 20,000 x g for 30 min at 4 °C. The supernatant was frozen at -80 °C. Using reagents and protocol from Life Technologies, NativePAGE Novex® Bis-Tris Gel System, G-250 dye was added to samples to a final concentration of 0.25%, and samples were run on a 3-12% polyacrylamide gel for 90 min at 150 V constant in the X-Cell *Sure Lock* Mini Cell gel apparatus by Life Technologies. Immunoblotting was performed with the X Cell II Blot Module (Invitrogen). The gel was soaked for 15 min in a 0.1% SDS solution with water and samples transferred to PVDF membrane in the X Cell transfer apparatus on ice for 1 h. Following transfer to the membrane, the membrane was fixed in 8% acetic acid and rinsed with methanol and dH_2_O. The primary antibody was mouse monoclonal anti-ATP5A antibody [15H4C4] by Abcam at a dilution of 1:3000 or 1:5000. The blocking buffer was StartingBlock (PBS) Blocking Buffer from Thermo Scientific with 0.1% Tween. The secondary antibody was goat anti-mouse IgG-HRP by Santa Cruz Biotechnology [sc-2005] at a dilution of 1:2500. Primary incubation occurred overnight, shaking at 4 °C and secondary incubation occurred at RT, shaking for 1 h. Antibodies were diluted with blocking buffer without Tween. Membranes were imaged and band intensity quantified using a Bio-Rad gel imager for chemiluminescence and ImageJ software. To determine the internal ratio of the dimer to monomer bands within each given lane, background was subtracted and band intensity measured on non-saturated blot images.

### Phylogenetic analysis

ATP synthase subunit paralogs were identified through FlyBase annotations and orthology tools [27, 70] supplemented with NCBI BLAST [71] and VectorBase [72] to search for ATP synthase subunits encoded by multiple paralogs in genomes outside the *Drosophila* genus. We used maximum likelihood phylogeny [73] to test if the proteins encoded by all paralogs cluster into two distinct groups. Clustering, tree drawing, and evolution rate analyses were performed in the MEGA5 suite [74, 75].
